# Improving anticancer effect of aPD-L1 through lowering neutrophil infiltration by PLAG in tumor implanted with MB49 mouse urothelial carcinoma

**DOI:** 10.1186/s12885-022-09815-7

**Published:** 2022-07-04

**Authors:** Guen Tae Kim, Eun Young Kim, Su-Hyun Shin, Hyowon Lee, Se Hee Lee, Ki-Young Sohn, Jae Wha Kim

**Affiliations:** 1 Enzychem Lifesciences, 10F aT Center 27 Gangnam-daero, Seoul, South Korea; 2 Division of Systems Biology and Bioengineering, Cell Factory Research Center, Korea Research Institute of Bioscience and Biotechnology (KRIBB), 125 Kwahak-ro, Daejeon, South Korea

**Keywords:** PLAG, Urothelial carcinoma, Anti-PD-L1, Neutrophil-to-lymphocyte ratio

## Abstract

**Background:**

The PD-L1 antibody is an immune checkpoint inhibitor (ICI) attracting attention. The third-generation anticancer drug has been proven to be very effective due to fewer side effects and higher tumor-specific reactions than conventional anticancer drugs. However, as tumors produce additional resistance in the host immune system, the effectiveness of ICI is gradually weakening. Therefore, it is very important to develop a combination therapy that increases the anticancer effect of ICI by removing anticancer resistance factors present around the tumor.

**Methods:**

The syngeneic model was used (*n* = 6) to investigate the enhanced anti-tumor effect of PD-L1 antibody with the addition of PLAG. MB49 murine urothelial cancer cells were implanted into the C57BL/6 mice subcutaneously. PLAG at different dosages (50/100 mpk) was daily administered orally for another 4 weeks with or without 5 mpk PD-L1 antibody (10F.9G2). PD-L1 antibody was delivered via IP injection once a week.

**Results:**

The aPD-L1 monotherapy group inhibited tumor growth of 56% compared to the positive group, while the PLAG and aPD-L1 co-treatment inhibited by 89%. PLAG treatment effectively reduced neutrophils infiltrating localized in tumor and converted to a tumor microenvironment with anti-tumor effective T-cells. PLAG increased tumor infiltration of CD8 positive cytotoxic T-cell populations while effectively inhibiting the infiltration of neoplastic T-cells such as CD4/FoxP3. Eventually, neutrophil-induced tumor ICI resistance was resolved by restoring the neutrophil-to-lymphocyte ratio to the normal range. In addition, regulation of cytokine and chemokine factors that inhibit neutrophil infiltration and increase the killing activity of cytotoxic T cells was observed in the tumors of mice treated with PLAG + aPD-L1.

**Conclusions:**

PLAG effectively turned the tumor-promoting microenvironment into a tumor-suppressing microenvironment. As a molecule that increases the anti-tumor effectiveness of aPD-L1, PLAG has the potential to be an essential and effective ICI co-therapeutic agent.

## Background

PD-L1 antibody is an immune checkpoint inhibitor (ICI) that inhibits tumors and tumor growth by blocking the ability of the tumor to avoid the host immune response. Tumor-specific expression of PD-L1 induces death of T-cells by binding to PD-1 of cytotoxic T lymphocytes. T cells can be maintained by blocking the binding of PD-L1 and PD-1. ICIs allow host T lymphocytes to attack tumors by interfering with the initial signaling pathway of tumor-specific immune evasion mechanisms [1–5]. However, it has recently been shown that PD-L1, specifically expressed in tumor cells, is also expressed in specific immune cells [6–8]. This may be a primary factor of ICI resistance and may reduce the anti-tumor efficacy of cytotoxic T cells. High expression of PD-L1 in tumor-infiltrating neutrophils (TINs) hinders the anti-tumor effectiveness of ICI treatment. The number of neutrophils increases extensively in tumor tissue, and PD-L1-expressing neutrophils interact with T lymphocytes to induce death and reduce the number of T cells [9–13]. For this reason, a high neutrophil-to-lymphocyte ratio (NLR) was frequently observed in patients with low effectiveness of ICI treatment and poor prognosis [14–17].

In addition to the decreased efficacy of ICI therapy, excessive TIN is a major cause of tumor growth [18–21]. Activated neutrophils express factors, such as elastase and myeloperoxidase (MPO), that stimulate specific receptors in tumor cells and activate tumor growth-related signaling pathways to facilitate tumor progression [22–26]. Moreover, active neutrophils increase the expression of MMPs, which promote the migration of tumor cells from the primary tumor site to the blood [27, 28] contributing to the early stages of tumor metastasis [29–31]. Therefore, reducing the number of TINs in tumor tissue is critical to maximizing the effectiveness of ICI therapy and tumor removal.

In this paper, we tested the synergistic anti-tumor effects of PLAG and ICI combination therapy. As a basic logic for combination therapy, PLAG lowers neutrophil infiltration in tumor tissue and increases cytotoxic T-cells, and ICI treatment enhances the activity of cytotoxic T-cells for tumor eradication. The combination therapy of PLAG and ICI inhibited tumor growth compared to each treatment group. This treatment effectively inhibited the excessive neutrophil infiltration in the tumor microenvironment, restored NLR to an average level, and increased the activity of cytotoxic T-lymphocytes. PLAG has a pivotal role in creating an environment for tumor suppression through effectively controlling immune cell activity and movement and reducing tumor growth factors expressed in tumor tissue recruited immune cells.

PLAG may be a highly effective anticancer drug because it eliminates the tumor microenvironment that hinders the efficacy of ICI, thereby increasing the killing of the tumor. PLAG and ICI combination therapy for tumor elimination can give hope to these cancer patients.

## Methods

### Test substance (PLAG) synthesis and manufacture

PLAG was manufactured and provided by the New Drug Production Headquarters, a GMP facility of Enzychem Lifesciences Corporation (Jecheon-si, South Korea). PLAG was stored according to the manufacturer’s instructions.

### Cell culture

MB49 murine urothelial cancer cells were obtained from the CMD Millipore corporation (Millipore, MD, USA). Both types of cells were grown in Dulbecco’s modified Eagle medium (DMEM; WelGENE, Seoul, Korea) containing 10% fetal bovine serum (HyClone, MA, USA) and 1% antibiotics (100 mg/L streptomycins, 100 U/mL penicillin) at 37 °C in a 5% CO_2_ atmosphere.

### Tumor implantation (syngeneic implantation)

Five-week-old male C57BL/6 mice were obtained from NARA biotech (Yong-in, South Korea) and housed in sterile filter-topped cages. The animals (*n* = 6 for each treatment group) were anesthetized using isoflurane and put in a position of right lateral decubitus. A total of 1 × 10^5^ MB49 cells in a solution containing 70 µL culture medium and 30 µL Matrigel (BD Biosciences, NJ, USA) were subcutaneously injected on the right side-thick using a 29-G needle permanently attached to a 0.5-mL insulin syringe (Becton Dickinson, NJ, USA). The mice were then allowed to rest on a heating carpet until fully recovered. Starting 4 days after implantation of cells, the mice were given daily oral doses of 50 or 100 mpk PLAG (*n* = 6 mice per group) with or without 5 mpk anti-PD-L1 once a week. A negative control group (*n* = 6 mice) was left untreated. Tumor burden was calculated every 3 days after implantation. The animals were sacrificed 5 weeks after implantation and perfused with PBS. The tumors were extracted and fixed with 10% formaldehyde. Hematoxylin and eosin (H&E) and immunohistochemical (IHC) staining was performed on the tissue sections to survey the tissue morphology. All animal experiments were approved by the IACUC, Korea Research Institute of Bioscience & Biotechnology (approval number: KRIBB-AEC-19219).

### anti-PD-L1 delivery

Anti-PD-L1 (clone; 10F.9G2) were purchased from BioXcell (BioXcell, MA, USA). The reagents were prepared according to the manufacturer's protocol and refrigerated until used. The delivery of the aPD-L1 was performed using the IP injection method, and the dose was injected at 17:30 every Tuesday. aPD-L1 was treated with 5 mg/kg/mice.

### FACS analysis

The extracted tumor was released into a single cell using a 40 µm-mash strainer. Whole blood and tumor were combined with the fluorochrome-conjugated specific antibody at room temperature for 30 min. After washing the samples twice using a FACS buffer, add a 1 × lysing solution (BD Biosciences, NJ, USA) and reaction for 15 min with slow agitation. Samples were washed twice and resuspension in the FACS buffer for analysis. Single cell was sorted by FACS versa and data analysis was using a FlowJo (FlowJo, LLC. OR. USA).

### Completer blood count (CBC) analysis

Hematopoietic analysis of the test mice was performed using a complete blood counts (CBC) analyzer (Mindray, chenzhen, China). Whole-body blood was used for cardiac hemorrhage and stored in a cube coated with EDTA until hemocyte analysis. For secreted proteins analysis in blood, serum was separated by centrifugation at 6,000 rpm for 10 min in a 4 °C.

### ELISA

The levels of secreted proteins in the mouse plasma were analyzed by factor-specific ELISA according to the manufacturer's protocol (R&D Systems, MN, USA). The absorbance was measured at 450 nm using an EMax Endpoint ELISA microplate reader (Molecular Devices Corporation, CA, USA).

### Immunohistochemistry (IHC) staining

Tissue specimens from the mice were fixed in 10% formaldehyde, embedded in paraffin, and sectioned into 5 µm slices. The sections were treated with 3% H_2_O_2_ for 10 min to block endogenous peroxidase activity and then blocked with bovine serum albumin. Then, the sections were washed in PBS and incubated with specific antibody overnight at 4 °C. Negative controls were incubated with the primary normal serum IgG for the species from which the primary antibody was obtained.

### Statistics

The data were analyzed using One-way ANOVA (Prism 9, GraphPad Software, CA, USA). *P* < 0.05 was considered statistically significant.

## Results

### PLAG and aPD-1 co-treatment enhanced the anti-tumor effects in MB49 urothelial cancer implanted mice

**Fig. 1:**
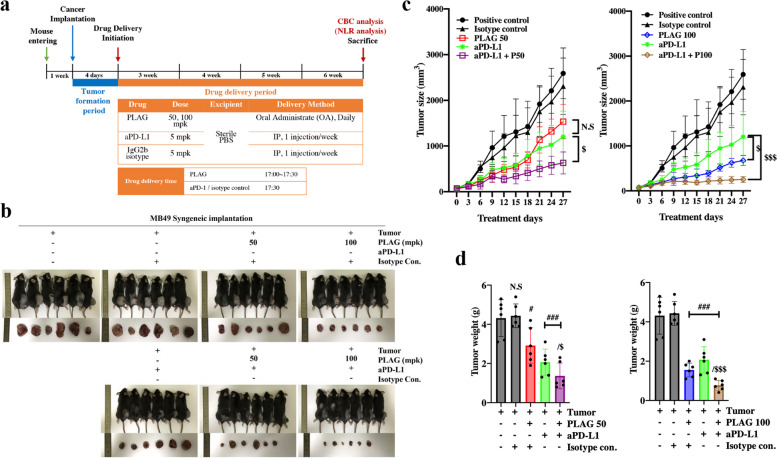
Inhibition of cancer progression by PLAG and aPD-L1 co-treatment in a MB49 urothelial cancer cell-implanted syngeneic mouse model. **a** Experimental design to evaluate the effect of PLAG + aPD-L1 co-treatment on tumor progression. **b** Tumor burden form and tumor size were determined in tumor-bearing mice treated with PLAG + aPD-L1 on the day of sacrifice. **c** Analysis of the increase in tumor size in each group based on measurements made at 3 d intervals. **d** Tumor weight was measured in tumor-bearing mice co-treated with PLAG + aPD-L1 on the day of sacrifice. Compared with the tumor only group: #*P* < 0.05, ##*P* < 0.01, ###*P* < 0.001; compared with the aPD-L1-only treatment group: $*P* < 0.05, $$$*P* < 0.001 (*n* = 6 for each experiment). N.S, not significant. Mean ± SD

The inhibitory effect of PLAG treatment on tumor growth in a mouse animal model of MB49 urothelial cancer was verified quantitatively. PLAG was administered to the mice daily for 4 weeks, and 5 mpk aPD-L1 was IP injected weekly (Fig. 1a). MB49 tumors are not known to be very sensitive to aPD-L1 therapy, thus an increased tumor burden was observed at week 4 after implantation. In the PLAG and aPD-L1 co-treatment group, tumor growth was retarded significantly (Fig. 1b). Tumor sizes were measured up to 4 weeks after tumor implantation. The PLAG alone treatment group had a 61% inhibitory effect on tumor growth when compared to the positive control group. The aPD-L1 alone treatment group had a 56% inhibitory effect on tumor growth when compared to the positive control group. In the 50 mpk PLAG + aPD-L1 co-treatment group, the inhibitory effect on tumor growth inhibitory was 48%, and in the 100 mpk PLAG + aPD-L1 co-treatment group, the inhibitory effect on tumor growth was 75% when compared to the aPD-L1 single treatment group (Fig. 1c). The weight of the tumor was measured on the day of sacrifice. In the aPD-L1 only treated group, tumor weight decreased by 48% compared to the positive control group. In the 100 mpk PLAG and co-treatment group, tumor weight decreased by 54% compared to the aPD-L1 only treated group. In the PLAG-only treatment group, tumor weight decreased by 55% compared to the positive control group (Fig. 1d).

### Increased circulating neutrophils during tumor growth were returned to normal levels in PLAG and aPD-L1 treated mice

**Fig. 2:**
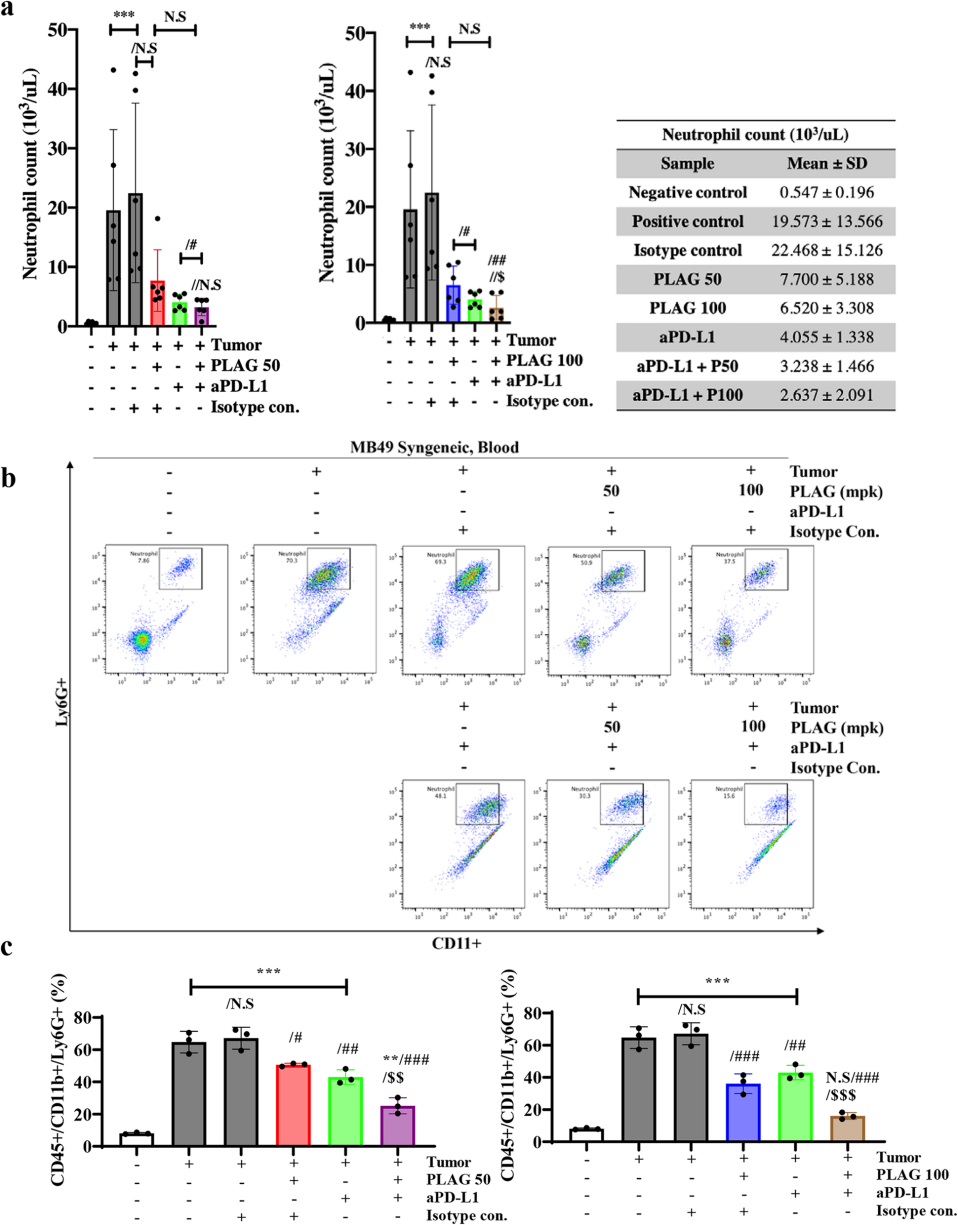
Neutrophils decreased in MB49 tumor-bearing mice treated with PLAG. **a** The number of neutrophils in the blood of tumor-bearing mice treated with PLAG were analyzed by CBC. Compared with the negative control: ***P < 0.001; compared with the positive control: #*P* < 0.05, ##*P* < 0.01; compared with the aPD-L1 only treatment group: $*P* < 0.05 (*n* = 6 for each experiment). N.S, not significant. Mean ± SD. **b** Among CD45^+^ leukocytes, Ly6G^+^ neutrophils were counted in the blood of tumor-bearing mice treated with PLAG + aPD-L1. Ly6G^+^ and CD11b^+^ cells were sorted by FACS. **c** Ly6G^+^ and CD11b^+^cells were counted in in the blood of tumor-bearing mice treated with PLAG. Compared with the negative control: ***P < 0.001; compared with the tumor only: #*P* < 0.05, ##*P* < 0.01, ###*P* < 0.001; compared with the aPD-L1-only treatment group: $$*P* < 0.01, $$$*P* < 0.001 (*n* = 3 for each experiment). N.S, not significant. Mean ± SD

Neutrophils in the blood were counted by CBC analyzer, and neutrophils with CD11b^ + ^/Ly6G^ +^ were quantitatively analyzed by FACS. The CBC analyzer calculated about 500/μl neutrophils in the blood of tumor-free normal mice. Total neutrophils increased to 20,000 neutrophils/μl during tumor growth and decreased to 7,700 and 6,500 neutrophils/μl in mice treated with 50 mpk and 100 mpk PLAG, respectively. Neutrophil levels in mice co-treated with PLAG and aPD-L1 decreased to 3,000/μl. (Fig. 2a). Among CD11b^ +^ myeloid cells, the number of Ly6G^ + ^neutrophils in the blood were counted. Similar to the total neutrophil reduction, the number of Ly6G^ +^ neutrophils decreased significantly upon PLAG treatment. CD11b^ +^ /Ly6G^ +^ cells were < 10% in normal mice and increased to 60% in tumor-bearing mice. CD11b^ +^ /Ly6G^ +^ cells decreased to < 40% in PLAG-treated mice and < 20% in PLAG + aPD-L1 co-treated mice. These data show that the circulating CD11b^ +^ /Ly6G^ + ^neutrophils were lowered effectively upon PLAG + aPD-L1 co-treatment (Fig. 2b,c).

### Decreased circulating lymphocytes during tumor growth were returned to normal levels in PLAG and aPD-L1 treated mice

**Fig. 3:**
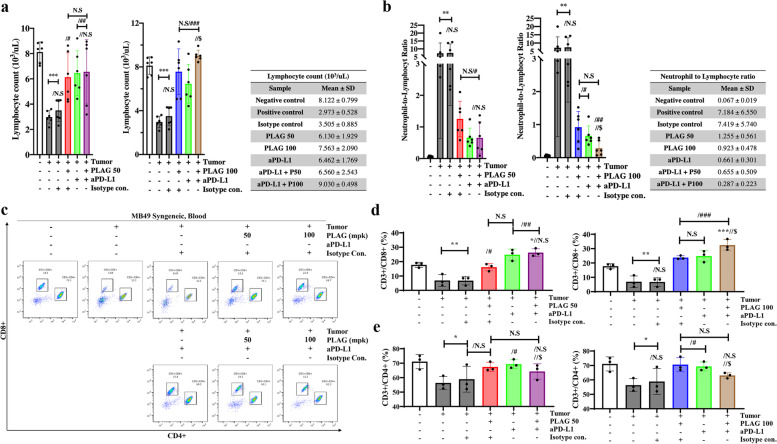
The reduced lymphocyte population in tumor-bearing mice was restored upon PLAG treatment. **a** The number of lymphocytes in the PLAG treatment group were counted by CBC. Compared with the negative control: ***P < 0.001; compared with the tumor only: #*P* < 0.05, ##*P* < 0.01, ###*P* < 0.001; compared with the aPD-L1 only treatment group: $*P* < 0.05 (*n* = 6 for each experiment). N.S, not significant. Mean ± SD. **b** The NLR in the blood of tumor-bearing mice treated with PLAG treatment was determined. Compared with the negative control: **P < 0.01; compared with the tumor only: #*P* < 0.05, ##*P* < 0.01; compared with the aPD-L1 only treatment group: $*P* < 0.05 (*n* = 6 for each experiment). N.S, not significant. Mean ± SD. **c** T cell populations in the blood of tumor-bearing mice treated with PLAG were evaluated. Among CD3^+^ cells, CD4^+^ cells and CD8^+^ cells were counted by FACS. **d**,** e** Blood CD4^+^ and CD8^+^ cells recovered from mice treated with PLAG were analyzed. Compared with the negative control: *P < 0.05, **P < 0.01; compared with the tumor only: #*P* < 0.05, ##*P* < 0.01, ###*P* < 0.001; compared with the aPD-L1-only treatment group: $$*P* < 0.01, $$$*P* < 0.001 (*n* = 3 for each experiment). N.S, not significant. Mean ± SD

Lymphocytes in the blood were counted by CBC, and lymphocytes with CD3^ +^ /CD4^ +^ or CD3^ +^ /CD8^ +^ were quantitatively analyzed through FACS. The CBC analyzer counted 8,000 lymphocytes/μl in the blood of tumor-free normal mice. Total lymphocytes decreased to 3,000 lymphocytes/μl during tumor growth and increased to 6,000/μL and 7,000/μl in mice treated with 50 mpk and 100 mpk PLAG, respectively. Lymphocyte levels in mice co-treated with PLAG + aPD-L1 increased to 8,000/μl similar to the negative control (Fig. 3a). The number of lymphocytes in the blood was classified as T-cell marker CD3^ +^ and analyzed quantitatively among lymphocyte cells. Like overall lymphocyte restoration, Similar to overall lymphocyte restoration, CD3^ +^ /CD4^ +^ and CD3^ +^ /CD8^ +^ lymphocyte levels were restored to normal levels upon PLAG treatment. CD3^ +^ /CD4^ +^ and CD3^ +^ /CD8^ +^ lymphocytes were at 70% and 20%, respectively, in normal mice and decreased to 50% and 5%, respectively, in mice with tumors. CD3^ +^ /CD4^ +^ and CD3^ +^ /CD8^ +^ levels recovered to normal at 70% and 20%, respectively, in PLAG-treated mice. In PLAG + aPDL-1 co-treated mice, CD3^ +^ /CD8 ^+^ lymphocytes recovered to > 30%. These data showed that circulating CD3^ +^ /CD8^ +^ lymphocytes increased significantly upon PLAG + aPD-L1 co-treatment (Fig. 3c-e).

PLAG treatment effectively restored the high blood NLR back to normal in tumor-bearing mice. In mice with tumors, circulating lymphocytes decreased while neutrophils increased. However, increased neutrophils were reduced in mice treated with PLAG, and decreased lymphocytes were restored in the blood. As a result of this experiment, in tumor-bearing mice, the NLR was ≥ 7, and in mice co-treated with PLAG + aPD-L1, the NLR was ≤ 1, which is similar to the NLR in normal mice (Fig. 3b.

### Tumor-infiltrating neutrophils (TINs) significantly decreased in PLAG + aPD-L1co-treated mice

**Fig. 4:**
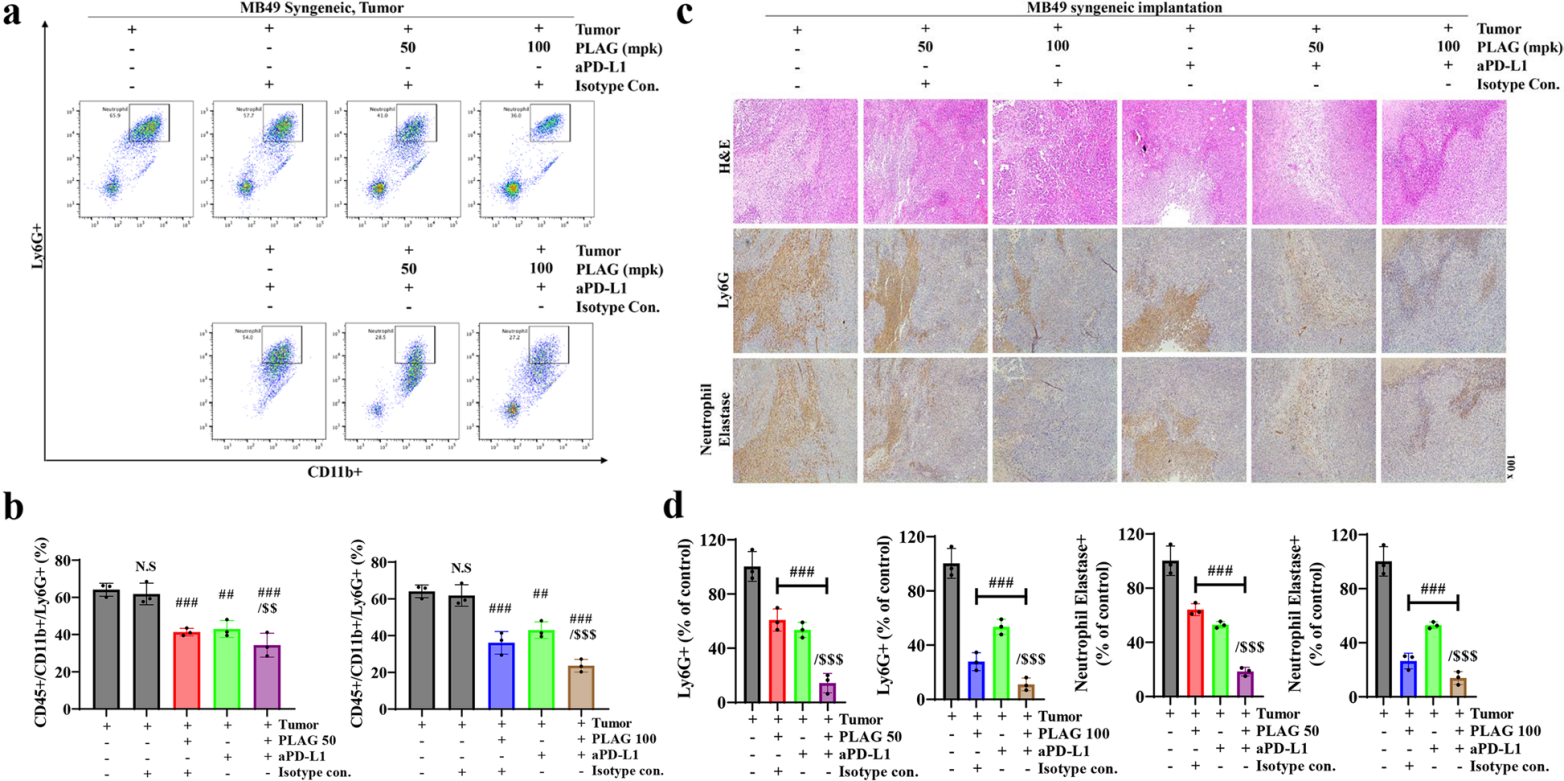
TINs were analyzed in tumor tissue from mice treated with PLAG.** a** Ly6G^+^ neutrophils in tumor tissue from mice treated with PLAG were counted. Among CD45^+^ leukocytes, Ly6G^+^ and CD11b^+^ cells were sorted by FACS. **b** Ly6G^+^ and CD11b^+^ TINs were analyzed in tumor tissue from mice treated with PLAG. Compared with the tumor only: #*P* < 0.05, ##*P* < 0.01, ###*P* < 0.001; compared with the aPD-L1 only treatment group: $*P* < 0.05, $$*P* < 0.01, $$$*P* < 0.001 (*n* = 3 for each experiment). N.S, not significant. Mean ± SD. **c** TINs in tumor tissue from mice treated with PLAG were confirmed by IHC staining with anti-Ly6G and anti-neutrophil elastase. **d** DAB-positive cells in tumor tissue stained with anti-Ly6G and anti-neutrophil elastase were analyzed. Compared with the tumor only: #*P* < 0.05, ##*P* < 0.01, ###*P* < 0.001; compared with the aPD-L1-only treatment group: $*P* < 0.05, $$*P* < 0.01, $$$*P* < 0.001 (*n* = 3 for each experiment). N.S, not significant. Mean ± SD

Neutrophils that infiltrate excessively into tumor lesions create a tumor microenvironment (TME) that contributes to tumor growth. CD11b^ +^ myeloid cells in tumor lesions of MB49 cell-implanted mice and Ly6G^ +^ neutrophils were evaluated by FACS analysis and IHC staining with neutrophil elastase and Ly6G antibodies. FACS analysis showed that Ly6G^ +^ neutrophils among CD11b^ +^ myeloid cells increased to 60% in MB49-implanted mice and decreased to 30% in tumor-bearing mice treated with PLAG. Ly6G^ +^ neutrophils were reduced to 20% PLAG + aPD-L1 co-treated mice but only reduced to 50% in anti-PD-L1-only-treated mice (Fig. 4a,b). These results confirmed that TIN decreased to normal levels in PLAG + aPD-L1 co-treated mice.

In addition, comparative analysis of the number of activated neutrophils by immunostaining with neutrophil elastase and anti-PD-L1 antibodies showed that the number of activated neutrophils reduced to 50% and 30% for the aPD-L1 only and PLAG only treatment groups, respectively. The number of activated neutrophils reduced to 10% in the PLAG + aPD-L1 co-treatment group (Fig. 4c,d). FACS and IHC staining analyses confirmed that TINs in TMEs were reduced effectively upon PLAG + aPD-L1 co-treatment.

### CD8.^+^ T lymphocyte populations increased in the tumor lesions of PLAG + aPD-L1co-treated mice

**Fig. 5:**
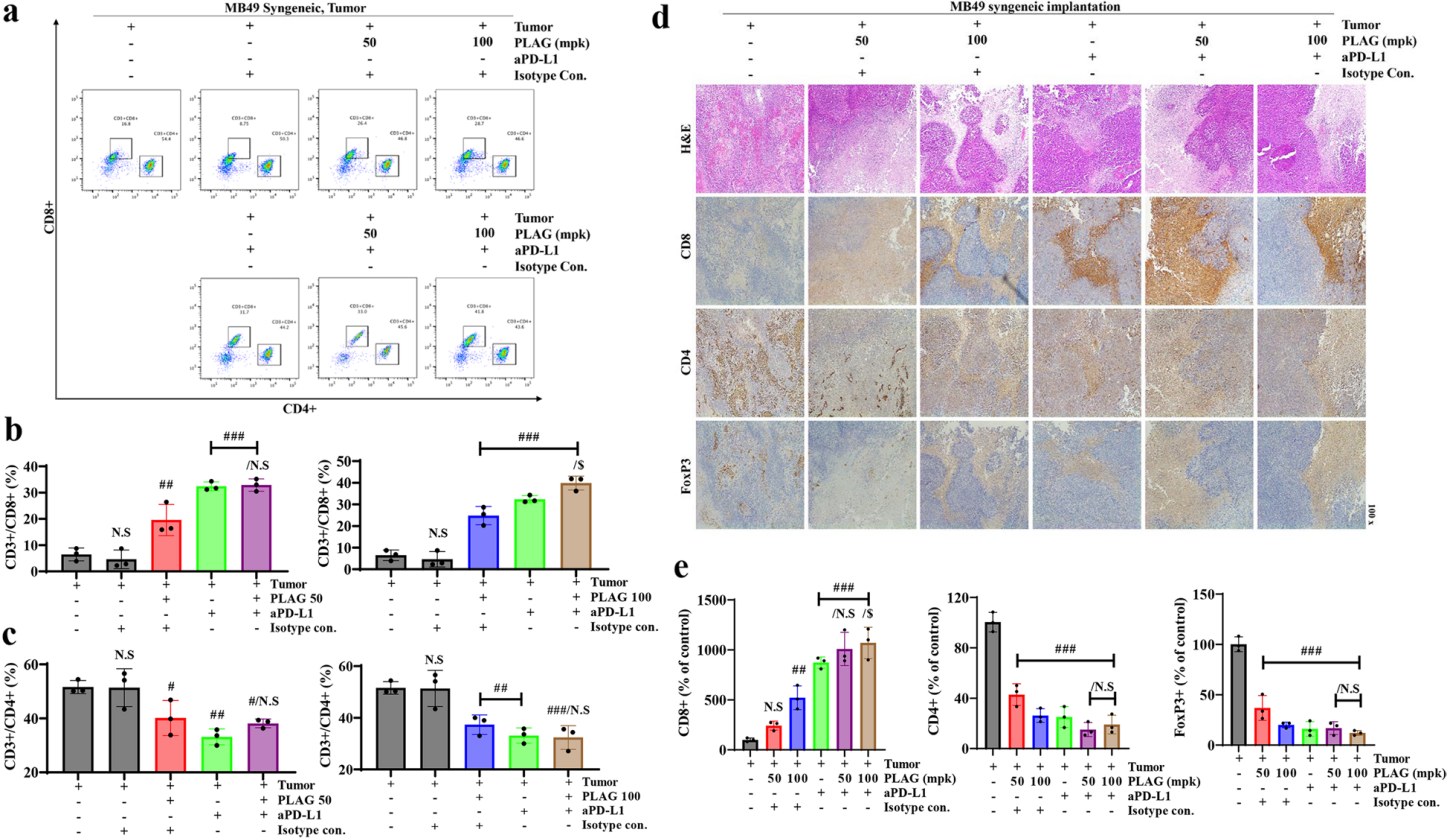
Analysis of T cell populations in tumor tissue from mice treated with PLAG. **a** T cell populations in tumor tissue from mice treated with PLAG were analyzed. Among CD3^+^ cells, CD4^+^ cells and CD8^+^ cells were counted by FACS. **b**,**c** Tumor-infiltrating CD3^+^/CD4^+^ cells and CD3^+^/CD8^+^ cells were analyzed in tumor tissue from mice treated with PLAG. Compared with the tumor only: #*P* < 0.05, ##*P* < 0.01, ###*P* < 0.001; compared with the aPD-L1 only treatment group: $*P* < 0.05 (*n* = 3 for each experiment). N.S, not significant. Mean ± SD. **d** Tumor-infiltrating T cells in tumor tissue from mice treated with PLAG were confirmed by IHC staining with anti-CD8, anti-CD4, and anti-FoxP3 antibodies. **e** DAB-positive cells in tumor tissue stained with anti-CD8, anti-CD4, and anti-FoxP3 were analyzed. Compared with the tumor only: #*P* < 0.05, ##*P* < 0.01, ###*P* < 0.001; compared with the aPD-L1-only treatment group: $*P* < 0.05, $$*P* < 0.01, $$$*P* < 0.001 (each experiment *n* = 3 for each experiment). N.S, not significant. Mean ± SD

The recruitment of CD8^ +^ cytotoxic T lymphocytes in tumor tissue is expected to inhibit tumor growth significantly. Therefore, among CD3^ +^ T cells, the numbers of CD3^ +^ /CD8^ +^ cytotoxic T cells and CD3^ +^ /CD4^ +^ helper T cells in tumor lesions of MB49 cell-implanted mice were evaluated by FACS and IHC analyses using antibodies to CD8, CD4, and FoxP3. FACS analysis showed that 50% of the T cells recruited to MB49 tumor-implanted tissue were CD4^ +^ helper T cells and < 10% were CD8^ +^ lymphocytes. Upon treatment with PLAG, the number of CD4^ +^ T cells recruited decreased to 30%. In mice co-treated with PLAG + aPD-L1, the number of CD8^ +^ T cells recruited to > 30% (Fig. 5a-c). IHC analysis showed that the number of CD8^ +^ T cells increased dramatically and the numbers of CD4^ +^ T cells and FoxP3^ +^ cells decreased significantly in mice co-treated with PLAG + aPD-L1 (Fig. 5d,e). FACS and IHC analyses showed that recruitment of CD3^ +^ /CD8^ +^ cytotoxic T cells, which suppress cancer cells, increased and that tumor-friendly CD3^ +^ /CD4^ +^ /FoxP3^ +^ T cells decreased upon co-treatment with PLAG + aPD-L1.

### PLAG and aPD-L1 inhibit tumor growth and regulate associated cytokines, chemokines, and tumor growth factors

Factors related to tumor growth and factors related to immune cell migration in the TME that are in the blood of mice with tumors and that are altered upon treatment with PLAG + aPD-L1 were analyzed by ELISA. The evaluated factors will provide scientific evidence to demonstrate the effectiveness of PLAG in transforming tumor-infiltrating immune cell populations for tumor suppression microenvironments.

**Fig. 6:**
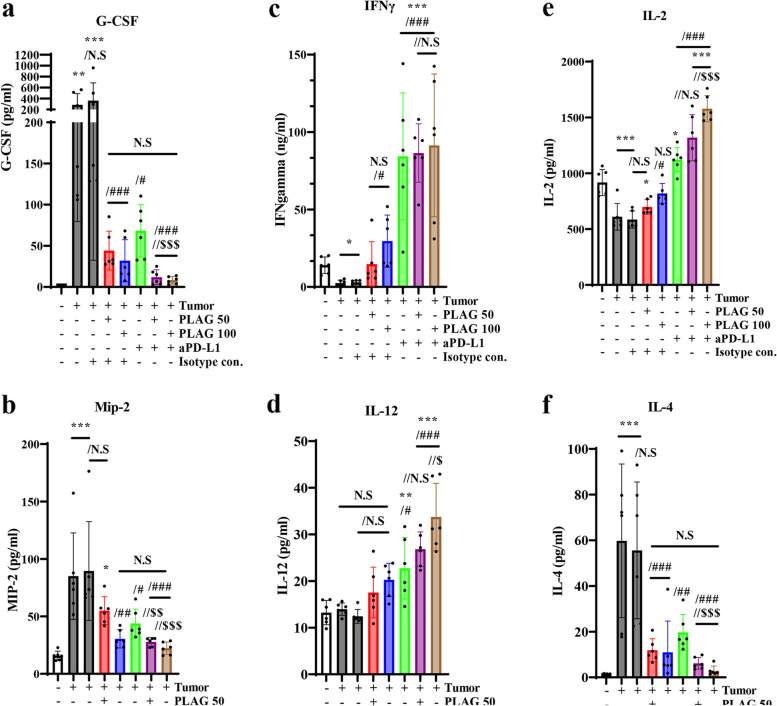
PLAG treatment alters the secretion of cytokines and chemokines in mice with MB49 tumors. **a**,**b** G-CSF, a cytokine that contributes to tumor growth, and MIP-2, a neutrophil migration factor, were evaluated in tumor-bearing mice treated with PLAG. **c**,**d** Th1, IFN-gamma, and IL-12 cytokines from tumor-bearing mice treated with PLAG were analyzed by ELISA. **e**,**f** T cell growth factor, IL-2, Th2, and IL-4 cytokines from tumor-bearing mice treated with PLAG were analyzed by ELISA. Compared with the negative control: *P < 0.05, **P < 0.01***P < 0.001; compared with the tumor only: #*P* < 0.05, ##*P* < 0.01, ###*P* < 0.001; compared with the aPD-L1-only treatment group: $*P* < 0.05, $$*P* < 0.01, $$$*P* < 0.001 (*n* = 6 for each experiment). N.S, not significant. Mean ± SD

Granulocyte stimulation factor (G-CSF), which is known to have been expressed in tumor cells for tumor growth, was highly expressed in MB49 implanted tumor tissue and effectively decreased to a level lower than that of normal mice in PLAG and aPD-L1 treated mice. Macrophage inflammatory protein-2 (MIP-2), a major chemotactic cytokine for neutrophil migration, significantly increased in MB49 implanted mice and effectively decreased in PLAG and aPD-L1 treated mice (Fig. 6a,b). Interferon-gamma (IFN-γ) and Interleukin-12 (IL-12), known as Th1-related cytokines serving as tumor suppression microenvironments, decreased slightly in MB49 implanted mice, but increased effectively in PLAG and aPD-L1 treated mice (Fig. 6c,d). Interleukin-2 (IL-2), known as T cell activator, is slightly decreased in MB49 transplanted mice but increased effectively in PLAG and aPD-L1 treated mice (Fig. 6e). Interleukin-4 (IL-4), known as Th2-related cytokines serving as tumor supportive microenvironments, increased in MB49 implanted mice, but significantly decreased in PLAG and aPD-L1 treated mice (Fig. 6f).

The results of cytokine analysis showed an increase in tumor-supportive cytokines in tumor implanted mice, but these were effectively reduced in PLAG and aPD-L1 treated mice. On the other hand, tumor-inhibiting cytokines were reduced in MB49 implanted mice, but significantly increased in PLAG and aPD-L1 treated mice.

## Discussion

ICI immunotherapy using PD-L1 antibody has fewer side effects and better results than the existing chemotherapy drugs, however the use of aPD-L1 has decreased efficacy and tumor resistance limitations due to various reasons [32–34], including activation of neutrophils and reduction of cytotoxic T-lymphocytes by neutrophils [35–37]. Therefore, regulating neutrophils could improve the efficacy of aPD-L1.

In this study, we showed the cooperative anti-tumor effect of co-treatment of aPD-L1 with PLAG, which reduced tumor-infiltrating neutrophils to normal levels. Quantitative analysis of inhibition of tumor growth in a mouse animal model implanted with MB49 and treated with PLAG and/or aPD-L1 showed a 45% increase in tumor growth inhibition in the co-treatment group compared to the aPD-L1 monotherapy group (Fig. 1c). In addition, tumor weight decreased 56% in the PLAG + aPD-L1 co-treatment group compared to the aPD-L1 monotherapy group (Fig. 1d). The increase in total neutrophils during tumor growth was reduced 72% in the PLAG + aPD-L1 co-treatment group compared to the PLAG and aPD-L1 monotherapy groups (Fig. 2). The number of cytotoxic T-lymphocytes, which inhibit tumor growth, recovered to an average level in tumor-bearing mice upon PLAG treatment. The number of cytotoxic T cells increased significantly in the PLAG + aPD-L1 combination therapy group compared with the aPD-L1 monotherapy group.

Because NLR is an effective prognostic marker of tumor treatment, we assessed NLR values for the treatment groups. In tumor-bearing mice co-treated with PLAG + aPD-L1, the NLR was reduced to the same level as in normal mice (Fig. 3). Tissue-infiltrating immune cells that make up the TME are important in controlling tumor growth. In general, TINs excessively activate signaling pathways associated with tumor growth and neutralize the anti-tumor activity of cytotoxic T cells [12, 17, 38, 39]. In fact, the ability of anticancer drugs to regulate the recruitment and activity of TINs may correlate with the anticancer activity of the drugs. In this study, the number of active neutrophils infiltrating the TME decreased upon PLAG + aPD-L1 co-treatment (Fig. 4a-c). In addition, neutrophil elastase is a factor that promotes cancer growth and metastasis. We showed that the level of neutrophil elastase decreased in cancer tissue in mice co-treated with PLAG + aPD-L1 (Fig. 4c,d). Tumor-infiltrating cytotoxic T lymphocytes also increased significantly in the tumor tissues of the PLAG-only and PLAG + aPD-L1 treatment groups (Fig. 5). In general, changes in the numbers of immune cells that infiltrate cancer tissues and changes in the TME are a result of cytokine expression [40–42]. One main factor that contributes to a TME that favors tumor development is an imbalanced Th1/Th2 cytokine ratio [43, 44]. Tumor-friendly TME includes higher Th2 type cytokines and fewer Th1 type cytokines. Reduced Th1 cytokines, IL-12 and IFNγ, in tumor implanted mice increased effectively in PLAG and aPD-L1 treated mice. Th2 cytokine, IL-4, increased in tumor-bearing mice but decreased dramatically in PLAG and aPD-L1 treated mice (Fig. 6).

In a previous study, we demonstrated the inhibitory effects of PLAG on tumors and tumor metastasis [45, 46]. In addition, in severe inflammatory diseases due to excessive neutrophil infiltration, PLAG treatment provided symptom relief by regulating the movement of neutrophils to inflammatory lesions [47–52]. The ability of PLAG to regulate neutrophil infiltration and activity in tumor tissue enables the anti-tumor effect of aPD-L1 to be maximized so that cancer patients can make a complete recovery.

## Conclusion

PLAG may be utilized for improving the efficacy of PD-L1 antibody on reducing the tumor burden at the devastating tumor microenvironment.

## Data Availability

All data generated or analyzed during this study are included in this published article.

## Abbreviations

PLAG: 1-Palmitoyl-2-limoleoyl-3-acetyl-roc-glycerol
ICI: Immune-checkpoint inhibitor
PD-L1: Programmed death-Ligand1
aPD-L1: Programmed death-Ligand1 antibody
PD-1: Programmed cell death protein-1
NLR: Neutrophil-to-lymphocyte ratio
TINs: Tumor infiltrating neutrophils
TME: Tumor microenvironment
CD: Cluster of differentiation
MPO: Myeloperoxidase
mpk: Milligram per kilogram
G-CSF: Granulocyte colony-stimulating factor
Mip-2: Macrophage inflammatory protein-2
IFNγ: Interferon gamma
IL: Interleukin
MMPs: Matrix metalloproteinases
